# Neuroinflammation and Neuronal Loss in the Hippocampus Are Associated with Immediate Posttraumatic Seizures and Corticosterone Elevation in Rats

**DOI:** 10.3390/ijms22115883

**Published:** 2021-05-30

**Authors:** Ilia G. Komoltsev, Stepan O. Frankevich, Natalia I. Shirobokova, Aleksandra A. Volkova, Mikhail V. Onufriev, Julia V. Moiseeva, Margarita R. Novikova, Natalia V. Gulyaeva

**Affiliations:** 1 Laboratory of Functional Biochemistry of the Nervous System, Institute of Higher Nervous Activity and Neurophysiology, Russian Academy of Sciences, 5A Butlerov Str., 117485 Moscow, Russia; komoltsev.ilia@ihna.ru (I.G.K.); stepan.frankevich@yandex.ru (S.O.F.); mr.brightside144@mail.ru (N.I.S.); aleksandra.al.volkova@gmail.com (A.A.V.); mikeonuf@ihna.ru (M.V.O.); jumois@ihna.ru (J.V.M.); mrnovikova@ihna.ru (M.R.N.); 2 Research and Clinical Center for Neuropsychiatry of Moscow Healthcare Department, 43 Donskaya Str., 115419 Moscow, Russia

**Keywords:** neuroinflammation, neurodegeneration, hippocampus, corticosterone, seizures, traumatic brain injury, epileptogenesis

## Abstract

Hippocampal damage after traumatic brain injury (TBI) is associated with late posttraumatic conditions, such as depression, cognitive decline and epilepsy. Mechanisms of selective hippocampal damage after TBI are not well understood. In this study, using rat TBI model (lateral fluid percussion cortical injury), we assessed potential association of immediate posttraumatic seizures and changes in corticosterone (CS) levels with neuroinflammation and neuronal cell loss in the hippocampus. Indices of distant hippocampal damage (neurodegeneration and neuroinflammation) were assessed using histological analysis (Nissl staining, Iba-1 immunohistochemical staining) and ELISA (IL-1β and CS) 1, 3, 7 and 14 days after TBI or sham operation in male Wistar rats (n = 146). IL-1β was elevated only in the ipsilateral hippocampus on day 1 after trauma. CS peak was detected on day 3 in blood, the ipsilateral and contralateral hippocampus. Neuronal cell loss in the hippocampus was demonstrated bilaterally; in the ipsilateral hippocampus it started earlier than in the contralateral. Microglial activation was evident in the hippocampus bilaterally on day 7 after TBI. The duration of immediate seizures correlated with CS elevation, levels of IL-1β and neuronal loss in the hippocampus. The data suggest potential association of immediate post-traumatic seizures with CS-dependent neuroinflammation-mediated distant hippocampal damage.

## 1. Introduction

Traumatic brain injury (TBI) is an important cause of neurological deficit and death [1]. TBI also leads to distant hippocampal damage, potentially resulting in post-traumatic epilepsy, depression and cognitive decline [2]. These complications often develop for years after the initial trauma. However, immediate post-traumatic seizures experienced by a number of patients may be a predictive behavioral correlate of acute brain damage, induced by excitatory amino acids release and its numerous effects. However, so far, no detailed analysis of either immediate posttraumatic seizures or their contribution to brain damage has been performed.

After initial period of trauma, a wide spectrum of pathological processes begins in the brain. Posttraumatic pathology, including selective distant hippocampal damage, is explained by both primary and secondary damage [3]. Excitotoxicity, edema, neuroinflammation and other factors are believed to underlie secondary neurodegeneration. The mechanisms and reasons for the selectivity of distant hippocampal damage after acute insults remain obscure. The involvement of the contralateral hippocampus in posttraumatic damage may indicate that not only local, but also systemic mechanisms contribute to hippocampal damage after TBI [4,5].

Systemic changes after TBI are also well documented. During stress response, glucocorticoids (GCs) bind to their receptors and modulate hippocampal function affecting numerous signaling and metabolic systems [6]. Different receptors of GCs are extensively expressed in the hippocampus and their distribution and properties may underlie the vulnerability of hippocampal neurons to different types of damage [7]. Specific aspects of GCs exposure critically determine the direction of their action, pro- or anti-inflammatory [8]. Acute GCs elevation is a vital physiological response to adverse stimuli, though very severe or chronic stress may be harmful and results in neuronal death and structural hippocampal damage. GCs also modulate glutamate receptors and may be involved in initial effects of TBI, including exacerbation of excitotoxicity [9]. This notion is supported by the fact that high initial GCs level and previous stress exacerbate hippocampal damage after brain insults in humans [10] and in rats [11].

It can be hypothesized that the selective vulnerability of the hippocampus to damage as well as distant and bilateral character of this damage after acute brain insults, in particular, TBI, may be at least partially explained by stress (inducing elevated CS) and excitotoxicity as a result of immediate posttraumatic seizures. In this study, we aimed to represent in detail immediate post-traumatic seizures in rats after fluid percussion brain cortical injury, characterize time course of CS changes, neuroinflammation and neurodegeneration in the ipsilateral and contralateral hippocampus during 2 weeks after trauma in rats and find potential associations between immediate seizures, CS elevation and distant hippocampal damage. Here, we report differences in time course of neuroinflammation and neurodegeneration in the ipsilateral and contralateral hippocampus and present evidence that immediate posttraumatic seizures may be associated with CS elevation, while both neuroinflammation and neuronal loss in the hippocampus are associated with immediate posttraumatic seizures and CS elevation.

## 2. Results

### 2.1. Characteristics of Immediate Posttraumatic Seizures in Rats

#### 2.1.1. Semiology of Immediate Posttraumatic Seizures

**Figure 1:**
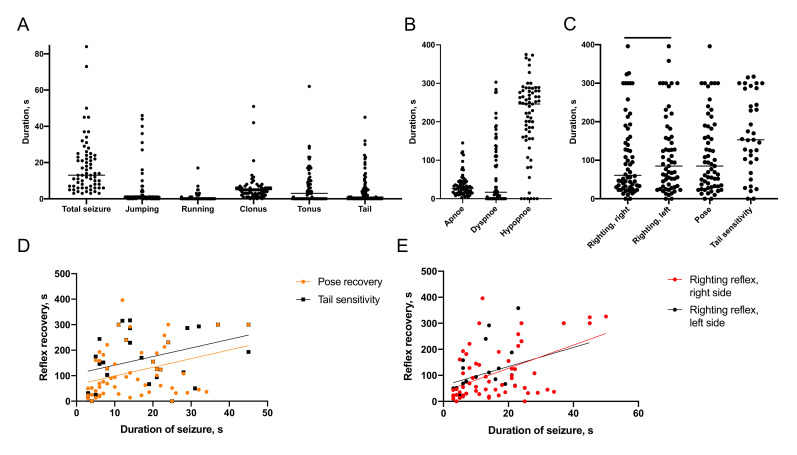
Seizure semiology. (**A**)—Duration of seizure episodes and their elements. (**B**)—Duration of respiratory disturbances. (**C**)—Duration of reflexes recovery. (**D**,**E**)—Correlations between the duration of immediate seizure episodes and recovery after TBI. (**A**)—Righting reflex recovery. (**B**)—Posture and tail sensitivity recovery. *p* < 0.05, Wilcoxon test (n = 67); for all correlations *p* < 0.05 (n = 67).

Post-traumatic seizures were detected in 100% of animals immediately after lateral fluid percussion (Figure 1). These seizures ranged from 3 to 84 s in duration, with an average of 18 ± 16 s. The seizures consisted of distinct forms of behavior in different sequences. In 37 of 67 experimental animals, seizures started with jumping associated with rapid extension of hind limbs. Another 11 animals displayed periods of jumping later during the seizure episode. These jumps varied in height, distance and direction. Among 48 animals exhibiting jumping, four animals demonstrated significant deviation to the left and five animals to the right. Running behavior was present either as a single period of movement or multiple periods of running, separated by other types of behavior. Cumulative duration of running ranged from 1 to 17 s, with an average of 4 ± 1 s. Among 13 animals exhibiting running, five animals moved straight, two deviated to the left and six to the right. Clonic seizures were observed in 63 of 67 animals; tonic seizures in 37 animals, seizures of the tail, or tail wriggling in 34 animals. The duration of clonic seizures ranged from 1 to 51 s, with an average of 6 ± 1 s; the duration of tonic seizures ranged from 1 to 62 s, with an average of 12 ± 2 s.

#### 2.1.2. Recovery of Breathing

The period of changing breathing patterns after the trauma can be described as a series of distinct periods. The standard sequence pattern is apnea→dyspnoe (nonrhythmic rare and deep breathing)→hypopnea (shallow amplitude breathing). Apnea, dyspnea and hypopnea were present in all injured animals and significantly varied in duration in different animals: 3 to 135 s with an average of 35 ± 4 s for apnea, 5 to 303 s with an average of 66 ± 11 s for dyspnea and 15 to 375 s with an average of 211 ± 12 s for hypopnea (Figure 1). Cyanosis of extremities potentially indicative of circulatory deficit was detected in 28% of experimental animals.

#### 2.1.3. Recovery of Reflexes

In general, the animals restored righting reflex and posture before tail sensitivity and then returned to active movement and exploration. The rats restored righting reflex 109 ± 13 s and 116 ± 13 s after TBI, right (ipsilateral) and left (contralateral) side, respectively (*p* < 0.05, Wilcoxon test). They restored the ability to maintain posture 116 ± 13 s after TBI. Tail sensitivity returned in 166 ± 17 s (Figure 1).

#### 2.1.4. Association of Immediate Seizures with Recovery after TBI

The duration of immediate seizure episodes correlated with the duration of apnea (r = 0.25, *p* < 0.05), dyspnea (r = 0.24, *p* = 0.05), recovery of righting reflexes (r = 0.37, *p* < 0.0001 for the right and r = 0.35, *p* < 0.005 for the left side), posture (r = 0.35, *p* < 0.01) and tail response (r = 0.40, *p* < 0.05; Figure 1).

In addition, we found correlations between the duration of several elements of seizures (Figure S1): duration of jumping correlated with that of running (r = 0.33, *p* < 0.005) and of clonic seizures (r = 0.40, *p* < 0.001). The duration of tail wriggling correlated with periods of tonic (r = 0.26, *p* < 0.05) and clonic (r = 0.47, *p* < 0.001) seizures.

#### 2.1.5. Predictors of Acute Mortality

We compared semiology of immediate seizures in deceased (10 rats, mortality rate 14.5%) and recovered rats. Prolonged seizures (16 ± 2 s in recovered vs. 30 ± 7 s in deceased animals, *p* < 0.05) and jumping (3 ± 1 s vs. 15 ± 5 s, *p* < 0.001), as well as longer duration of dyspnoe (46 ± 9 s vs. 177 ± 28 s, *p* < 0.0005), righting reflexes (98 ± 14 s vs. 174 ± 32 s for the right, *p* < 0.05 and 106 ± 14 s vs. 179 ± 36 s for the left side, *p* < 0.05) and posture recovery (106 ± 13 s vs. 179 ± 36 s, *p* < 0.05) were associated with acute mortality (Figure S2).

Cyanosis (an index of circulatory dysfunction) was associated with prolonged apnoe (30 ± 4 s vs. 47 ± 7 s, *p* < 0.05), righting reflexes (90 ± 14 s vs. 156 ± 26 s, *p* < 0.005, 98 ± 14 s vs. 168 ± 26 s, *p* < 0.05) and posture recovery (98 ± 14 s vs. 168 ± 26 s, *p* < 0.05). Notably, cyanosis occurred more frequently (6/4 vs. 13/44 rats, *p* < 0.05, Fisher exact test) in animals that did not survive.

### 2.2. Distant Hippocampal Damage

In this section, we present the time course of distant hippocampal damage (neuroinflammation and neurodegeneration) in the ipsi- and contralateral hemispheres.

#### 2.2.1. Neuroinflammation in the Hippocampus

**Figure 2:**
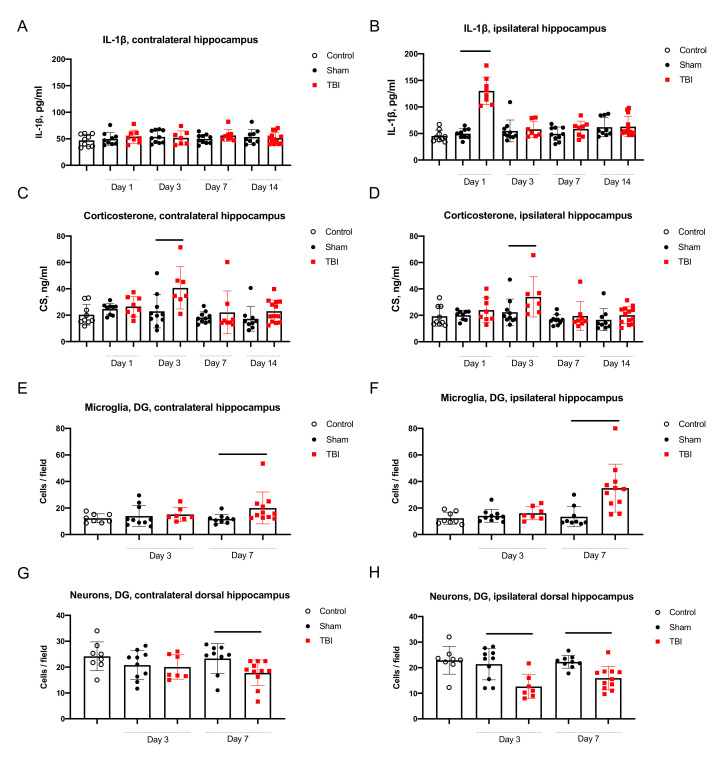
Neuroinflammation and neurodegeneration in the hippocampus. (**A**,**B**)—elevated IL-1β in the ipsilateral hippocampus on day 1 after TBI. (**C**,**D**)—Elevated CS in the ipsilateral and contralateral hippocampus on day 3 after TBI. (**E**,**F**)—Elevated microglial cell count in the hippocampus on day 7 in the ipsilateral and contralateral hippocampal DG. (**G**,**H**)—Decreased neuronal cell count in the ipsilateral hippocampal DG on day 3 and in the contralateral hippocampal DG on day 7 after TBI. *p* < 0.05, Mann-Whitney test.

Elevation of IL-1β was evident in the ipsilateral hippocampus as compared to sham operated rats 1 day after TBI (49.7 ± 3.2 pg/mL vs. 130.4 ± 9.2 pg/mL *p* < 0.001), but not in contralateral hippocampus (Figure 2). Levels of IL-1β in the ipsilateral and contralateral hippocampi demonstrated a positive correlation (r = 0.25, *p* < 0.05) when in all rats were taken in the analysis (Figure S3).

Unlike the asymmetrical IL-1β elevation, the morphology of microglial cells changed in both the ipsilateral and contralateral hippocampus. Microglial cells had thicker body and processes on day 3 and 7 after TBI (Figure S4). The number of microglial cells was higher in both the ipsilateral and contralateral hippocampi on day 7 (Figure 2). In the ipsilateral hippocampus the microglial cell count increased in the dentate gyrus (DG) (13.5 ± 2.5 cells in sham-operated rats vs. 35.0 ± 5.4, *p* < 0.005), CA3 (11.1 ± 1.7 vs. 19.4 ± 4.0, *p* = 0.07, statistical trend) and CA1 fields (12.3 ± 2.0 vs. 27.4 ± 4.0 cells, *p* < 0.005). In the contralateral hippocampus of rats with TBI microglial cell count was also augmented in the DG (11.8 ± 1.2 vs. 20.0 ± 3.6, *p* < 0.01), CA3 (8.2 ± 1.0 vs. 12.6 ± 1.2, *p* < 0.05) and CA1 fields (9.3 ± 1.0 vs. 15.0 ± 1.6, *p* < 0.01).

#### 2.2.2. Neurodegeneration in the Hippocampus

Average number of neurons in the DG, CA3 and CA1 hippocampal fields did not change on day 3 and 7 after TBI as compared to sham-operated rats. We compared neuronal cell count for the dorsal and ventral hippocampus separately, because of clearly distinct anatomy and different physiological role of these hippocampal parts. On day 3, the number of neurons in DG, polymorph layer of the ipsilateral dorsal hippocampus was lower in rats after TBI (21.4 ± 1.9 vs. 12.6 ± 1.8, *p* < 0.05). On day 7, the number of neurons in the ipsilateral hippocampal DG was lower (22.3 ± 0.8 vs. 15.9 ± 0.4, *p* < 0.005), however, the changes were also detected in the DG of contralateral dorsal hippocampus (23.3 ± 1.9 vs. 17.5 ± 1.5, *p* < 0.05; Figure 2, see also Figure S5). In the ventral hippocampus changes of neuronal cell count were not found.

#### 2.2.3. Corticosterone Elevation in Blood and the Hippocampus

**Figure 3:**
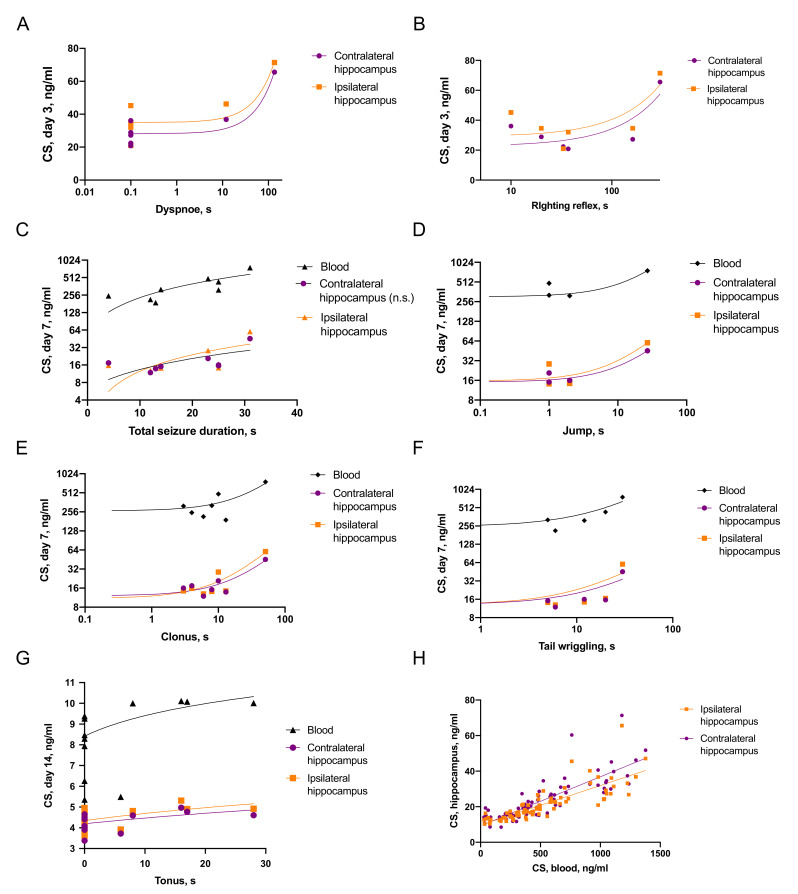
Associations between immediate seizures and CS elevation. (**A**,**B**)—Duration of dyspnoe and recovery of righting reflex correlates with CS level on day 3 (n = 7). (**C**–**F**)—Duration of immediate seizure elements correlates with CS level in blood and the hippocampus on day 7 after TBI (n = 8). (**G**)—Duration of tonus correlates with CS level in blood and the hippocampus on day 14 after TBI (n = 13). (**H**)—Correlations of CS levels in blood and the hippocampus (n = 81).

On day 1 CS elevation in blood was seen in several animals with TBI (deviation from mean was higher as compared to sham-operated rats), but it was not statistically significant between groups (Figure S6). On day 3, after TBI CS become elevated simultaneously in blood (543.9 ± 118.1 ng/mL vs. 964.1 ± 108.0 ng/mL, *p* < 0.05), as well as the ipsilateral and contralateral hippocampus (23.0 ± 4.0 ng/mL vs. 40.7 ± 6.0 ng/mL, *p* < 0.05 and 22.4 ± 3.2 ng/mL vs. 34.0 ± 5.7 ng/mL, *p* < 0.05, respectively; Figure 2). CS level in blood correlated with its level in the ipsilateral (r = 0.66, *p* < 0.001) and contralateral hippocampus (r = 0.68, *p* < 0.005; Figure 3).

### 2.3. Involvement of Immediate Seizure and Corticosterone Elevation in the Distant Hippocampal Damage

In this section, we analyze associations of immediate seizures and CS elevation with the distant hippocampal damage.

#### 2.3.1. Associations of Seizures and CS Increase

Elements of immediate seizures were associated with pronounced CS elevation (Figure 3). On day 3 after TBI the level of CS in the ipsilateral and contralateral hippocampus correlated with prolonged dyspnoe (r = 0.76, *p* < 0.05 and r = 0.87, *p* < 0.005) and righting reflex (r = 0.59, *p* = 0.07, statistical trend and r = 0.65, *p* = 0.05) immediately after TBI. On day 7 we revealed numerous correlations between blood CS, CS in the ipsilateral and contralateral hippocampus and elements of immediate posttraumatic seizures: total duration of seizures (r = 0.64, *p* < 0.05; r = 0.41, *p* = 0.09 and n.s., respectively), jumping (r = 0.72, *p* < 0.01; r = 0.91, *p* < 0.0001 and r = 0.94, *p* < 0.0001, respectively), clonic phase of seizure (r = 0.60, *p* < 0.05; r = 0.88, *p* < 0.005 and r = 0.89, *p* < 0.001) and tail wriggling (r = 0.59, *p* < 0.05; r = 0.43, *p* = 0.06 and r = 0.51, *p* < 0.05, respectively). On day 14 levels of CS in blood, the ipsilateral and contralateral hippocampus correlated with the duration of tonic phase of seizures (r = 0.54, *p* < 0.01; r = 0.38, *p* < 0.05 and r = 0.32, *p* = 0.05, respectively).

#### 2.3.2. Associations between Seizures and Distant Hippocampal Damage

**Figure 4:**
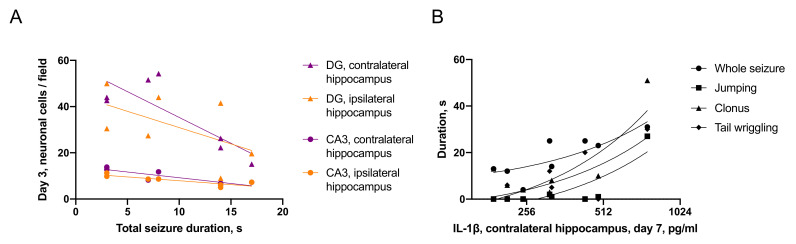
Associations between immediate seizures and distant hippocampal damage. (**A**)—Duration of immediate seizure correlates with neuronal cell loss in the hippocampus on day 3 after TBI (n = 7). (**B**)—Duration of immediate seizure elements correlates with the level of IL-1β in the contralateral hippocampus on day 7 after TBI (n = 8).

Total duration of seizure episode and its tonic phase negatively correlated with neuronal cell count in the ipsilateral hippocampal CA3 field (r = 0.76, *p* < 0.05; r = 0.73, *p* < 0.05), contralateral DG (r = 0.67, *p* < 0.05; r = 0.84, *p* < 0.01) and contralateral CA3 (r = 0.77, *p* < 0.01; r = 0.64, *p* < 0.05) on day 3 after TBI (Figure 4A). Furthermore, on day 7 IL-1β level in the contralateral hippocampus correlated with total duration of seizures (r = 0.51, *p* < 0.05), jumping (r = 0.88, *p* < 0.05), clonic phase of seizure (r = 0.67, *p* < 0.05) and tail wriggling (r = 0.83, *p* < 0.01; Figure 4B).

In rats, demonstrating jumping during immediate seizures the number of neurons in the contralateral hippocampus was lower in both DG (56 ± 9 vs. 29 ± 5, *p* < 0.05) and CA3 (12 ± 1 vs. 7 ± 1, *p* < 0.05), as compared to rats without jumping on day 7 after TBI.

#### 2.3.3. Associations between CS and Distant Hippocampal Damage

**Figure 5:**
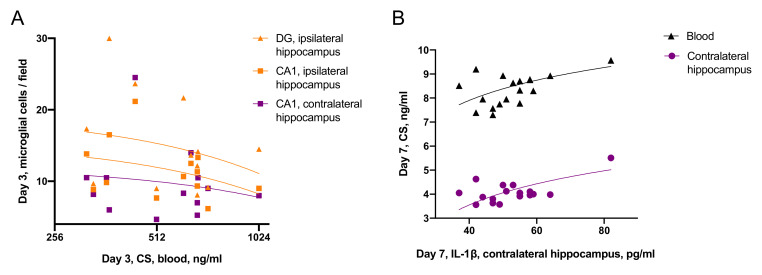
Time-dependent associations of CS and distant hippocampal damage. (**A**)—CS negatively correlates with microglial activation on day 3 after TBI (n = 7). (**B**)—CS positively correlates with IL-1β in the contralateral hippocampus on day 7 after TBI (n = 8).

The correlations between CS and neuroinflammatory response in the hippocampus were time-dependent and ambiguous (Figure 5). On day 3, blood CS level negatively correlated with the microglial cell count in the hippocampus, specifically in the ipsilateral DG (r = 0.52, *p* < 0.05) and CA3 (r = 0.58, *p* < 0.05) fields, as well as in the contralateral DG (r = 0.48, *p* = 0.06, statistical trend) and CA3 (r = 0.55, *p* < 0.05). These data may be related to an anti-inflammatory response to pronounced CS elevation. In contrast, on day 7 after TBI levels of IL-1β in the contralateral hippocampus positively correlated with CS in the same region (r = 0.65, *p* < 0.005) and in blood (r = 0.60, *p* < 0.01). This may reflect a pro-inflammatory effect CS, detected in the contralateral hippocampus later after TBI.

## 3. Discussion

In this study, we presented a detailed descriptions of immediate seizures following TBI in rats. We revealed different time course of neuroinflammation and neurodegeneration in the ipsilateral and contralateral hippocampus. In the TBI model used, the development of these pathological processes most probably involves both local and systemic mechanisms. Next, we compared characteristics of immediate seizure episodes with levels of CS, neuroinflammation and neurodegeneration in the hippocampus. We found that immediate posttraumatic seizures are associated with CS elevation and neurodegeneration in the hippocampus. We also found out putative pro- and anti-inflammatory effects of CS on neuroinflammation in the hippocampus.

### 3.1. TBI and Its Late Consequences

TBI is a prevalent cause of death and disability among a wide array of people of all ages and backgrounds across the population [12]. Limitations of existing epidemiological studies coupled with increasing diagnostic capacity in low-income areas where TBI is routinely under-reported suggest a further rise of incidence in the near future [13]. While deaths as a result of TBI have decreased from 50% to 25% in past 30 years, severe neurological disorders following TBI are becoming a common complication among survivors of head trauma [14]. According to current evidence, these changes have neuroinflammation and tissue damage at the core of their pathogenesis [15].

Human studies in this field have obvious limitations. Neuropathology of hippocampal damage in patients with mesial temporal lobe epilepsy, the most common type of posttraumatic epilepsy, is well characterized in humans and is highly reproducible in animal models of chronic seizures [16]. To understand early mechanisms of late posttraumatic pathology lateral fluid percussion brain injury in rats is widely used [3,17,18]. According to the reports of different groups, unprovoked seizures in the late period occur in 20–40% of rats [19,20,21,22]. Seizure types after TBI in rats include behavioral arrests, perioral automatisms, myoclonic seizures [21] and tonic-clonic seizures [23]. Cognitive impairment and elements of affective impairment in rats after lateral fluid percussion injury were also reported [24,25].

### 3.2. Seizure Semiology in Humans and in Animal Models

Clinical assessment of seizure semiology is a common technique used in medical practice [26]. When analyzing seizures in human patients, medical specialists focus on clinical manifestations of a seizure, breathing patterns, state of consciousness and reflexes [27]. Clinical manifestations of seizures in patients are incredibly diverse. These features can be reliably associated with definite pathological activity and serve as markers for specific types of epilepsy. Pathological changes in different functional cortical zones determine semiology of seizures, presence of consciousness, postictal confusion period [26]. Tonic or clonic components of seizure episode depend on frequency of discharge in the motor cortex [28]: if the rate of discharge is lower than the rate of muscle relaxation, clonic seizures occur, otherwise it results in tonic contraction. Inhibitory mechanisms may be involved in reduction of discharge frequency and influence semiology of acute seizures and its gradual changes. We believe that applying clinical view and detailed description of seizure episodes to animal models may provide valuable insights into pathological changes, including acute provoked seizures after TBI.

Analysing available data, we have realized that, though models of post-traumatic epilepsy appear well established, early seizures are rarely described. Several groups reported immediate post-traumatic seizures in injured animals [22,29], however, did not provide descriptions of these seizures. Many studies use the Racine Scale [30] and modified versions of this approach to assess seizures [31]. This scale classifies seizures by categories based on their apparent severity (from 0 to 5; mouth and face clonus, clonic jerks of one or two forelimbs, clonus, rearing and falling are considered). Racine scale provides a useful tool to assess severity of a seizure, but it lacks detailed assessment of specific features of seizure episode.

### 3.3. First Minutes after TBI: Immediate Posttraumatic Seizures and Reflex Recovery

Primary damage after TBI includes direct injury to brain tissue and mechanical disruption of blood–brain barrier. Acute injury is associated with rapid elevation of excitatory amino acids in extracellular space, increase in extracellular K+ and energy deficit. A change in the extracellular ion concentration reduces the excitability threshold of neurons and is further aggravated by their repetitive firing. Non-synaptic mechanisms of synchronization are associated with an increase in extracellular K+, a decrease in Ca2+, changes in pH, accumulation of glutamate and delayed utilization of glutamate and K+ by astrocytes [32], as well as the “ephaptic coupling” (excitation of neurons caused by the exchange of ions between the cells) [33]. Synaptic mechanisms of neuronal synchronization include wide convergence of glutamatergic neurons (for example, in the CA3 field, in the DG of the hippocampus) and a “chain” excitation reaction [32]. Thus, acute injury provides a basis for neuronal synchronization and symptomatic seizures.

Circulatory changes are among major physiological consequences of TBI. Mean arterial blood pressure elevates immediately after TBI and then returns to baseline within 5 min; it may also decrease in the cases of severe TBI. Transient hypoxemia (apnea, cyanosis and decrease of PaO_2_ in blood) was also described in acute phase of TBI [3,17]. Circulatory failure is believed to increase mortality after TBI in rats. According to our data, cyanosis of extremities (as a marker of circulatory deficit) was associated with prolonged apnea, extended reflexes recovery and increased mortality. These data are consistent with previously reported results [3]. The involvement of hypoxemia in hippocampal neuronal loss is discussed; however, hypoxic damage does not seem to be a main cause for selective distant hippocampal damage [34]. In our study, we also did not reveal differences between biochemical and morphological characteristics of hippocampal damage in animals with and without cyanosis of extremities.

In this study, we presented a detailed description of rat behavior immediately after lateral fluid percussion brain injury. We analyzed incidence and duration of different elements of acute symptomatic seizure episodes. All seizures were bilateral with tonic and clonic components, lateralization during seizure being relatively rare. Though immediate seizures were detected in all rats, the semiology varied in different. Its variability may indirectly reflect different degrees of acute brain damage, including excessive release of excitatory amino acids and its numerous extensive effects in the whole brain. We found positive correlations between specific elements of seizure episodes (jumping, running and clonic seizures), suggesting that these semiology patterns may reflect a continuity of events. For example, the duration of tail wriggling correlated with that of both tonic and clonic seizures and may represent its continuation.

We found that seizure duration correlated with recovery of righting reflexes, posture and tail response after trauma (within minutes after TBI). The control of breathing, circulation, righting reflexes and pose involves brainstem nucleus, while purposeful reaction on tail stimulation involves many levels, including cortex [35,36,37]. Our data confirm that TBI affects the brain extensively, including brainstem (circulatory and breathing dysfunction, righting reflex). A gradual recovery from TBI was observed (from subcortical structures to cortex and from the ipsilateral to contralateral hemisphere, according to righting reflex asymmetry). Importantly, in this timescale (minutes after trauma) oedema or inflammation are just beginning [38] and are unlikely involved in the rapid loss and recovery of consciousness. Excessive excitatory and inhibitory neuronal activity and spreading depression might be a more probable explanation of these phenomena [39].

### 3.4. Hours and Days after TBI: Mechanisms of Distant Hippocampal Damage

In addition to the formation of a cortical lesion [3], TBI induces a secondary, distant neuronal death and glial activation in the CA3 field and DG gyrus of the hippocampus [40]; changes, though less pronounced, are also evident in the contralateral hippocampus [41,42]. GABA-ergic neurons (parvalbumin, calretinin, somatostatin and neuropeptide Y—immunoreactive) in the hilus are among most vulnerable populations of hippocampal cells. Histological changes after TBI are analogous to damage in the hippocampus after pharmacologically induced seizures [43,44]. Acute excessive release of glutamate and aspartate leads to cell death via activation of NMDA receptors, entry of Na+, Ca2+ into the cell, release of K+ [3], apoptosis and necrosis of neurons as a result of excitotoxicity [45,46,47].

In this study, we showed different time course of neuronal cell loss in the hippocampus: in the ipsilateral hemisphere it was detected on day 3, while in the contralateral hippocampus neuronal cell loss was detected only on day 7 after TBI. IL-1β accumulation on day 1 after TBI was also asymmetrical and may be involved in subsequent ipsilateral hippocampal neurodegeneration. Neuronal cell loss in the ipsilateral and contralateral hippocampus may result from the realization of various mechanisms (local and systemic); putative machinery involved in neurodegeneration will be discussed below.

Why hippocampal neurons of both hemispheres are selectively vulnerable to posttraumatic damage? We hypothesize that effects of circulating GCs subsequently accumulating in the hippocampus are pivotal for bilateral hippocampal damage after TBI. It is well known that GCs regulates stress response and their receptors are widely expressed in the hippocampus. Functions of GCs in the hippocampus are mediated by high-affinity mineralocorticoid and low-affinity glucocorticoid receptors, activated by high levels of GCs. Furthermore, two types of these receptors exist: cytoplasmic/nuclear receptors, associated with slow genomic action and non-genomic membrane-associated receptors rapidly altering excitatory neurotransmission [7]. Effects of GCs on neuronal excitability mediated by mineralocorticoid and glucocorticoid receptors are dose-sensitive: glutamatergic synaptic transmission is increased by low doses of CS acting at membrane mineralocorticoid receptors (a rapid stress response) and decreased by higher doses acting at glucocorticoid receptors (recovery from stressful situation) [7,48]. GCs regulate neuronal excitability, neuroinflammation and may play a key role in hippocampal sensitivity to initial excitotoxic damage and further secondary neuronal death [49]. Effects of GCs on seizures in rats are realized via modulation of excitability and enhancement of seizure susceptibility [50]. Moreover, GCs increase the vulnerability of neurons to excitotoxicity [9]. Mechanisms of these effects of GCs include suppression of glucose transport, glutamate reuptake by astrocytes, alternation of Ca2+ homeostasis and suppression of neurotrophic factors production [51,52]. GCs exhibit either pro- or anti-inflammatory properties, depending on specific features of the definite situation, including the degree and duration of GCs exposure, factors and characteristics of the injury and spatial/temporal aspects [53]. It is suggested that in the hippocampus GCs may act as pro-inflammatory agents [8,53,54]. Importantly, increased level of GCs before a pro-inflammatory stimulus has an additional pro-inflammatory effect [55]. This effect is believed to underlie acute neurodegeneration after acute brain insults [4]. Cytokines, pivotal pro-inflammatory molecules, modify functioning of glutamate and GABAergic receptors, inhibit the uptake of glutamate by astrocytes, disrupt the function of voltage-dependent ion channels, induce an increase in the extracellular concentration of K+, leading to neuronal hypersynchronization and further neurodegeneration [56].

In this study, CS elevation was found in both hemispheres on day 3 after TBI, as a response to physiological stress. We detected multiple correlations between blood and hippocampal CS levels and duration of immediate seizures. In rats with prolonged seizures, CS was higher on days 3, 7 and 14. CS elevation on day 3 negatively correlated with microglial cells density in the hippocampus, reflecting a well-known anti-inflammatory effect of GCs [53] (IL-1β and microglial cells density on day 3 in TBI rats were not elevated). On day 7, microglial activation was detected in both hemispheres and it was accompanied by neuronal loss in the contralateral hippocampus. Thus, at least in the contralateral hippocampus CS may exert its anti-inflammatory effect: CS positively correlated with IL-1β accumulation, reflecting possible pro-inflammatory action of this glucocorticoid. We also report correlations between immediate seizures, neuronal loss on day 3 after TBI in both hemispheres and IL-1β accumulation in the contralateral hippocampus on day 7 after TBI. Both acute excitotoxicity immediately after seizures and delayed effects of CS may be responsible for these correlations.

An association between distant hippocampal damage and hippocampal excitability was shown experimentally. Neuronal loss in the hippocampal DG is accompanied by and correlated with an increase of hippocampal neurons excitability after perforant path stimulation one week after TBI [34]. In our previous study, we reported that spontaneously generated high-amplitude epileptiform hippocampal spike occurrence correlates with increase of microglial cell density and neuronal loss in the hippocampal DG 7 days after TBI [57].

### 3.5. Months and Years after TBI: Late Posttraumatic Pathology

Further progressive changes in the hippocampus are closely associated with changes in neuroplasticity [58], chronic neuroinflammation, astrocyte disfunction and neurogenesis. As a result of persistent neuroinflammation [59] and increased excitability of neurons, there is an increase in neurogenesis in the DG of the hippocampus, aberrant migration of new generated cells and abnormal integration into neural ensembles. These cells are also characterized by hyperexcitability and contribute to the development of spontaneous convulsive activity [60].

## 4. Materials and Methods

### 4.1. Environment and Housing

The study was performed on 146 male Wistar rats aged 6 months and weighting 379 ± 3 g. The animals were housed in acrylic containers, with spruce shavings provided as bedding and food and water access ad libitum. All experiments with animals were performed in accordance with the EU Directive 2010/63/EU. The experimental protocol was approved by the Ethical Commission of the Institute of Higher Nervous Activity and Neurophysiology, Russian Academy of Sciences (protocol number 10, 10 December 2012). All efforts were made to minimize animal suffering.

Animals were randomly divided into 3 groups. Sixty-nine rats underwent a scull trepanation and lateral fluid-percussion brain injury (TBI group). Sixty rats underwent a scull trepanation without brain injury (Sham operation group). Another 18 rats formed the intact control group.

### 4.2. Experimental Design

We used lateral fluid-percussion injury (LFPI) in rats as a model of TBI [61]. This model is well-described and was chosen since it closely imitates conditions of TBI in human patients and allows precise calibration of applied force. All experimental animals in TBI and Sham operation groups were put under total inhalational anesthesia using 2% isoflurane. After scull exposure through a midline incision, a 3 mm wide trepanation aperture was created in the right parietal bone (AP = 3 mm, L = 3 mm). A plastic head from a Luer-type needle was attached to the aperture with acrylic glue to serve as a hub for applied pressure from LFPI device (Fluid Percussion Device with the PC-Based Pressure Measurement Unit, Model FP302, Richmond, VA, USA). Once the animals retained full consciousness after the operation, they were placed in a square Styrofoam box, 60 cm on each side with walls 40 cm high, to maintain the animal and protect it from additional trauma during a seizure. A LFPI (3.03 ± 0.03 atm) was delivered through the aperture. During a subsequent seizure and recovery period, the animals were tested for various reflexes and circulatory deficit, as described further. Video recordings of the moment of trauma, immediate seizures triggered by the trauma and following recovery period were made. After all recordings were completed, the animals were returned to their home cages.

**Figure 6:**
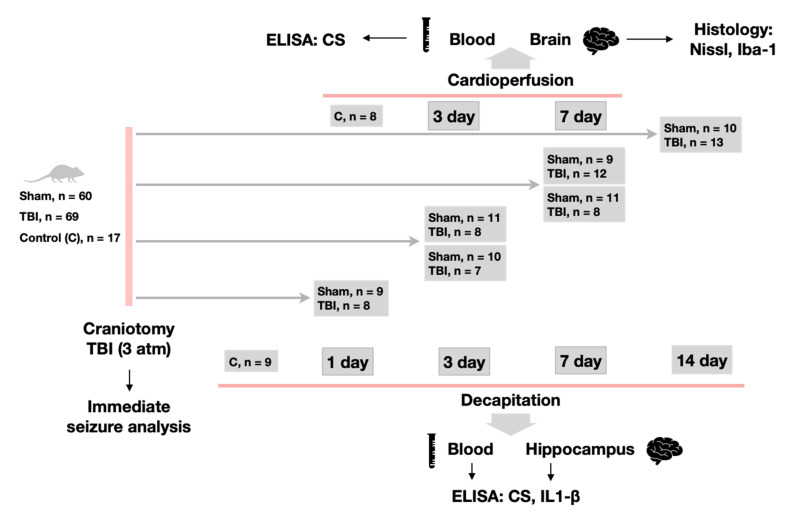
The design of the experiment.

Experimental animals remained in their cages and were sacrificed on days 1, 3, 7 and 14 (17, 17, 19 and 23 animals, respectively) for biochemical analysis and on days 3 and 7 for histology (19 and 21 rats, respectively). Control groups consisted of 9 animals for biochemistry and 8 for histology. The animals of the first group series (for biochemical analysis) were sacrificed by quick decapitation using a guillotine. Decapitation blood in these groups was sampled and centrifuged at 1500× *g* and 4 °C for 15 min to obtain serum. The brain was removed and after cooling in ice-cold saline, the hippocampus (ventral and dorsal parts) was isolated. Animals of the second group series (for histological analysis) were sacrificed under anesthesia through arterial perfusion with 4% solution of formaldehyde. Before sacrifice we sampled blood through a tail vein. After perfusion, the brain was fixed in the same solution (Figure 6).

### 4.3. Immediate Seizure Analysis

A visual analysis of all obtained recordings was performed. Each recording was divided into periods corresponding to behavioral repertoire. These periods were measured and a sequence of seizure events was produced for every animal. We noted whether a seizure started with a series of jumps and recorded lateralization of these jumps; measured total duration of seizure episode, duration and lateralization of running, duration of clonic and tonic seizures and convulsions of the tail, lateralization of clonic seizures. The duration of apnea, dyspnea and hypopnea were recorded and signs of cyanosis on extremities noticed. Righting reflex, ability to maintain stable quadrupedal position and sensitivity to pain were registered. When testing righting reflex, we tilted animals slightly to the right and left sides. If the rat was able to return back to its starting position, we registered it as return of right or left righting reflex accordingly. Pain sensitivity tests were conducted by firmly pinching the tip of rat’s tail.

### 4.4. Histological Analysis

Vibratome sections of 50 μm thickness were prepared from rat brains. Sections located 600 μm apart with coordinates between 2.1 and 5.8 mm from the bregma were selected for analysis. Sections were stained using Nissl method with Cresyl Violet dye. For the immunohistochemical staining (ionized calcium-binding adaptor molecule 1, Iba 1, a microglial marker), floating slices were washed in PBS and then rinsed in PBST (0.01-M PBS with 0.3% Triton X-100). The slices were incubated in a blocking solution (5% normal goat serum (MP Biomedicals, Irvine, CA, USA) in PBST) and then incubated overnight in the solution containing rabbit anti-Iba1 antibody (Wako, Neuss, Germany) at 4 °C. Then, the sections were thoroughly washed in PBST and incubated in the solution containing Alexa Fluor 488 goat antibody to rabbit IgG (1:500, Invitrogen, Carlsbad, CA, USA) for 2 h. Sections were mounted with ProLong Gold Antifade with DAPI (Invitrogen, Carlsbad, CA, USA) and coverslipped.

Microphotographs of sections stained by the Nissl method were made using a Keyence BZ-X700 microscope (Itasca, IL, USA) by immersion microscopy (magnification ×60). Four sections were selected for counting of the total number of neurons in the polymorphic layer of DG and CA1, CA3 areas of the hippocampus in both hemispheres. Microphotographs of immunohistochemically stained sections were made using a ZEISS (Oberkochen, Germany) Apotome microscopy (magnification ×20). The density of microglial and neuronal cells calculated as number of cells in 150 × 150 μm visual field, in the polymorph layers of the DG, CA1 and CA3 areas of the hippocampus. Further calculations of microglial cells were made using the ImageJ 1.52q program.

### 4.5. Biochemical Analysis

Tissue samples of the left and right hippocampus were homogenized in a Potter homogenizer in cold buffer in ×10 volume of the homogenization solution (PBS, 0.1% NP-40, protease inhibitor cocktail (Roshe, Basel, Switzerland)) with 10 strokes of the pestle at a rotation speed of 1000 rpm. Homogenate was centrifuged (16,900× *g*, 15 min, 4 °C) and the resulting supernatant was used to determine the level of corticosterone and proinflammatory cytokines by ELISA. Aliquots of the supernatants were stored at −80 °C. Enzyme-linked immunosorbent assay kits were used for measurements of corticosterone level in blood serum and in hippocampal supernatants (Corticosterone ELISA, DRG, Marburg, Germany), which detected both free and bound corticosterone by competitive ELISA. Proinflammatory cytokine IL-1β in the blood serum and hippocampus of rats was detected using enzyme immunoassay kits (Rat IL-1 beta/IL-1F2 Quantikine ELISA Kit, R&D Systems, Minneapolis, MN, USA) according to the manufacturer’s instructions.

### 4.6. Statistical Analysis

Statistical analysis was performed using STATISTICA 12 software (StatSoft, Tulsa, OK, USA). Visualization was made using Graph Prism 8. The Mann–Whitney U-test was used to compare independent variable (Sham vs. TBI groups). The Wilcoxon test was used for dependent variable. For continuous variables, Pearson’s correlations were calculated. All data are presented as mean ± SEM (standard error of mean).

## 5. Conclusions

- We report a first detailed description of seizure episodes immediately after lateral fluid percussion brain injury in rats and correlations between duration of immediate seizures and duration of reflexes recovery after TBI.
- IL-1β was elevated only in the ipsilateral hippocampus on day 1 after trauma. Microglial activation was evident in the ipsilateral and contralateral hippocampus 7 days after TBI. The duration of immediate seizures correlated with levels of IL-1β on day 7 after TBI.
- Neuronal cell loss was detected bilaterally, in the ipsilateral hippocampus it started earlier than in the contralateral. The duration of immediate seizures correlated with neuronal cell loss in the hippocampus on day 3 after TBI.
- CS was elevated on day 3 after TBI and the duration of immediate seizures correlated with CS levels.
- Effects of CS on neuroinflammation was time-dependent: on day 3 blood CS negatively correlated with microglial activation, while on day 7, blood CS positively correlated with IL-1β level in the contralateral hippocampus.

## Data Availability

Data available on the demand.

## Supplementary Materials

The following materials are available online at https://www.mdpi.com/article/10.3390/ijms22115883/s1.

## Abbreviations

CA1	cornu ammonis 1, hippocampal field
CA3	cornu ammonis 1, hippocampal field
CS	corticosterone
DG	dentate gyrus
ELISA	enzyme-linked immunosorbent assay
GABA	gamma-aminobutyric acid
GCs	glucocorticoids
Iba1	ionized calcium-binding adaptor molecule 1
IL-1β	interleukin-1β
LFPI	lateral fluid percussion injury
NMDA	N-methyl-D-aspartate
PBS	phosphate buffer saline
