# Supplementary material for: Neuroinflammation and Neuronal Loss in the Hippocampus Are Associated with Immediate Posttraumatic Seizures and Corticosterone Elevation in Rats
Source: Int J Mol Sci. 2021 May 30;22(11):5883. doi: 10.3390/ijms22115883 (PMC8198836; doi:10.3390/ijms22115883)
Supplement: Supplementary file 1 [file ijms-22-05883-s001.zip › ijms-1225574-supplementary.pdf]

Supplementary Materials

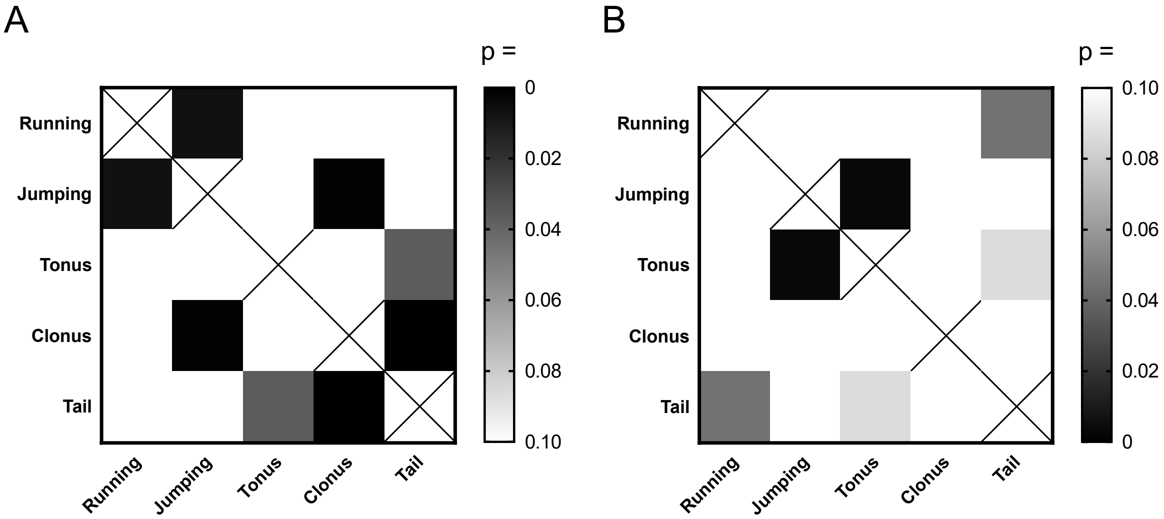

Figure S1. Correlations of immediate seizure episode elements. A – Correlations of immediate seizures elements duration. Duration of jumping correlated with duration of running and clonus. Duration of tail wriggling correlated with tonus and clonus. B – Representation of seizure episode elements (Fischer exact test). In rats with jumping tonic seizures appeared more frequently.

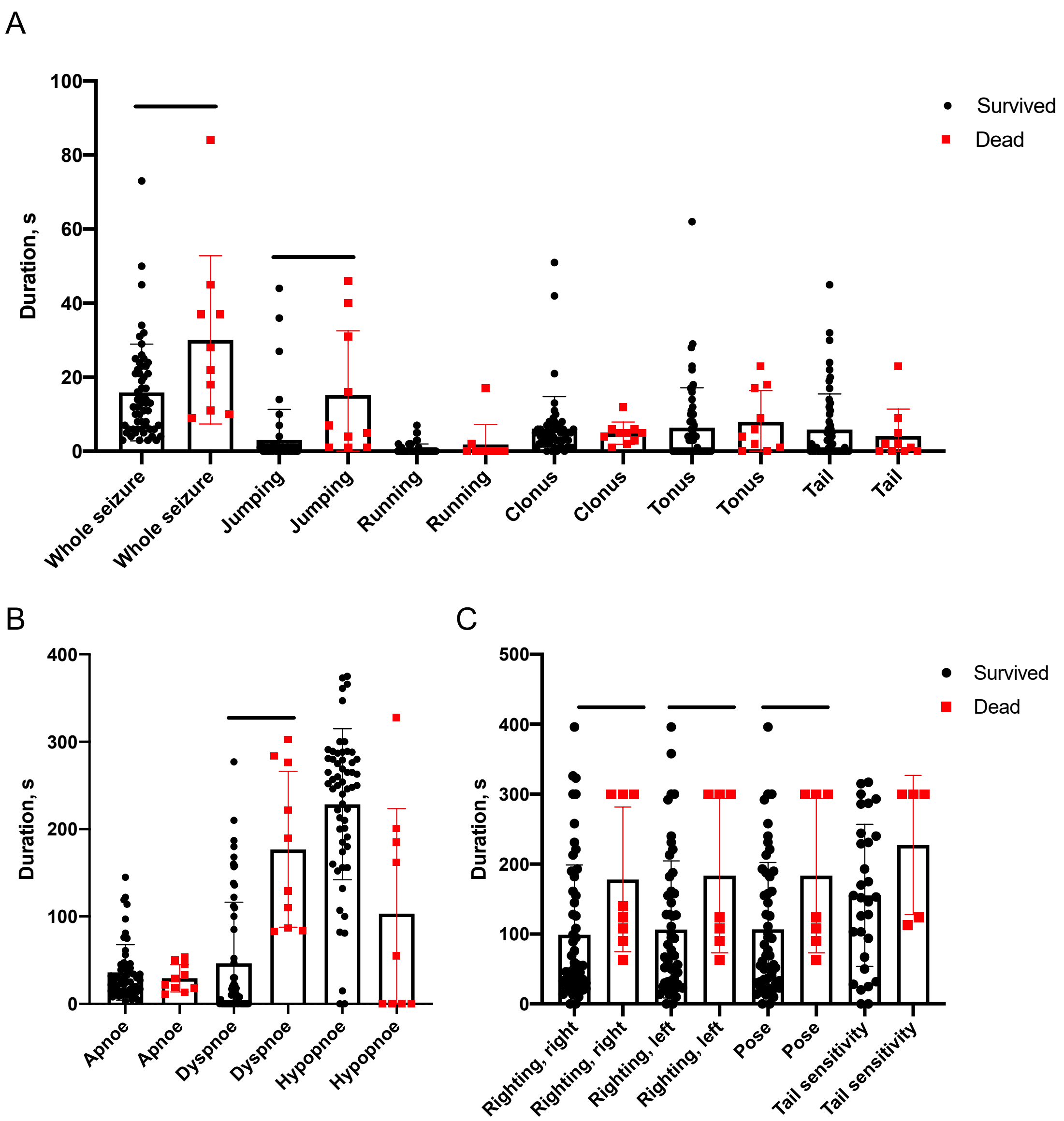

Figure S2. Mortality predictors. A – Longer duration of seizures and jumping was higher in animals which died. B – Dyspnoe was longer in rats which died. C – Recovery of reflexes were longer in rats which died. * - $p<0.05$, Mann-Whitney test.

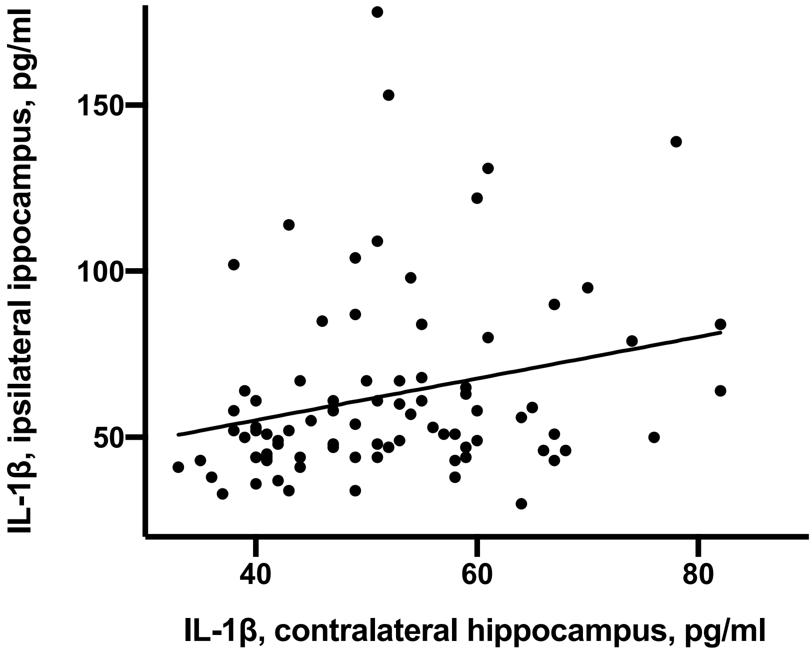

Figure S3. Correlation of IL-1β in the ipsilateral and contralateral hippocampus.

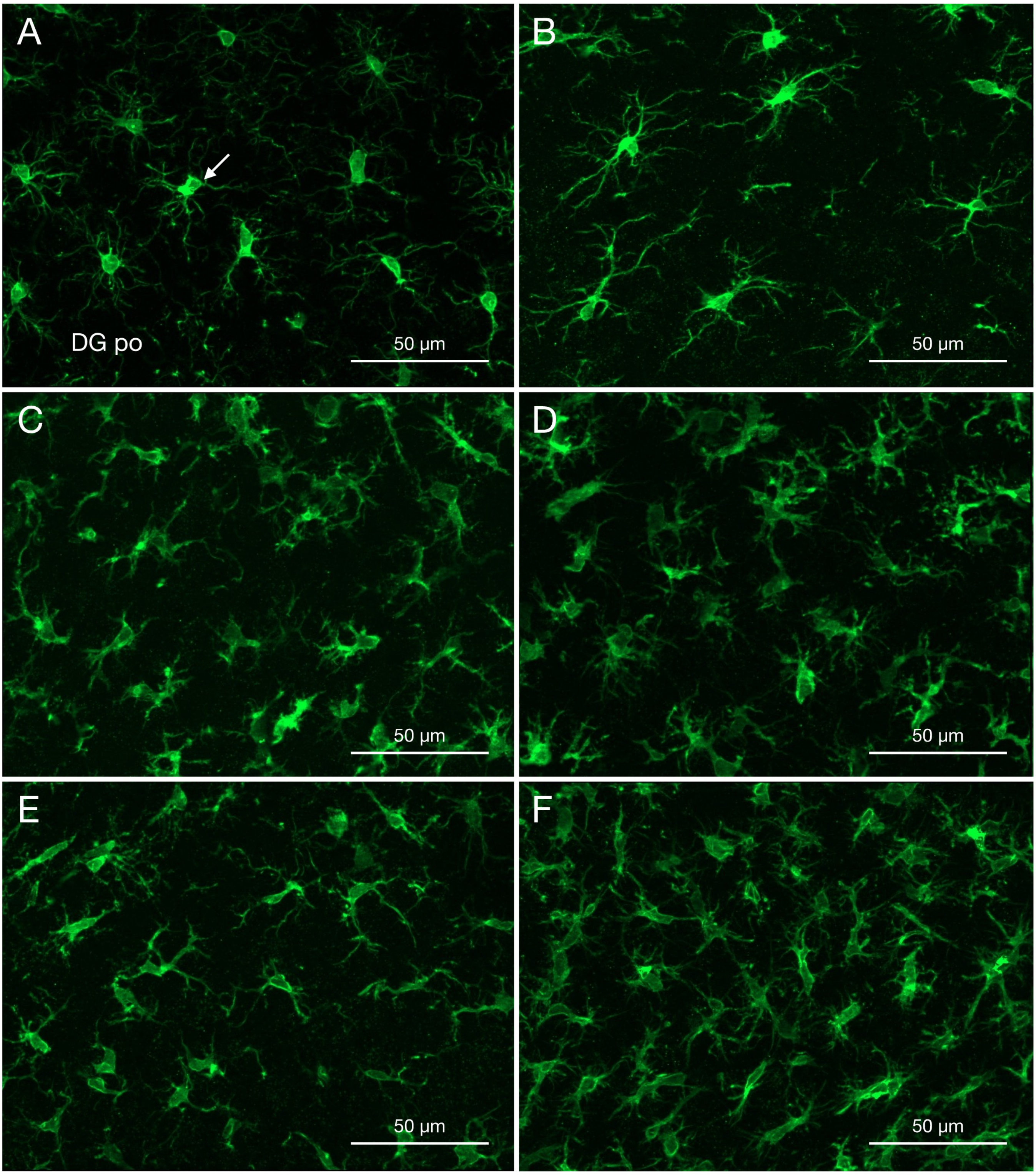

Figure S4. Microglial changes in the hippocampus. A – control, right hemisphere. B – Sham operated rats, day 7 after craniotomy. C, D – contralateral and ipsilateral hippocampus (bilateral changes in microglial cell morphology), day 3 after TBI. E, F - contralateral and ipsilateral hippocampus, day 7 after TBI (increasing in microglial cell density s bilaterally). Anti-Iba-1 staining. DG – dentate gyrus, po – polymorph layer. The arrow shows microglial cell.

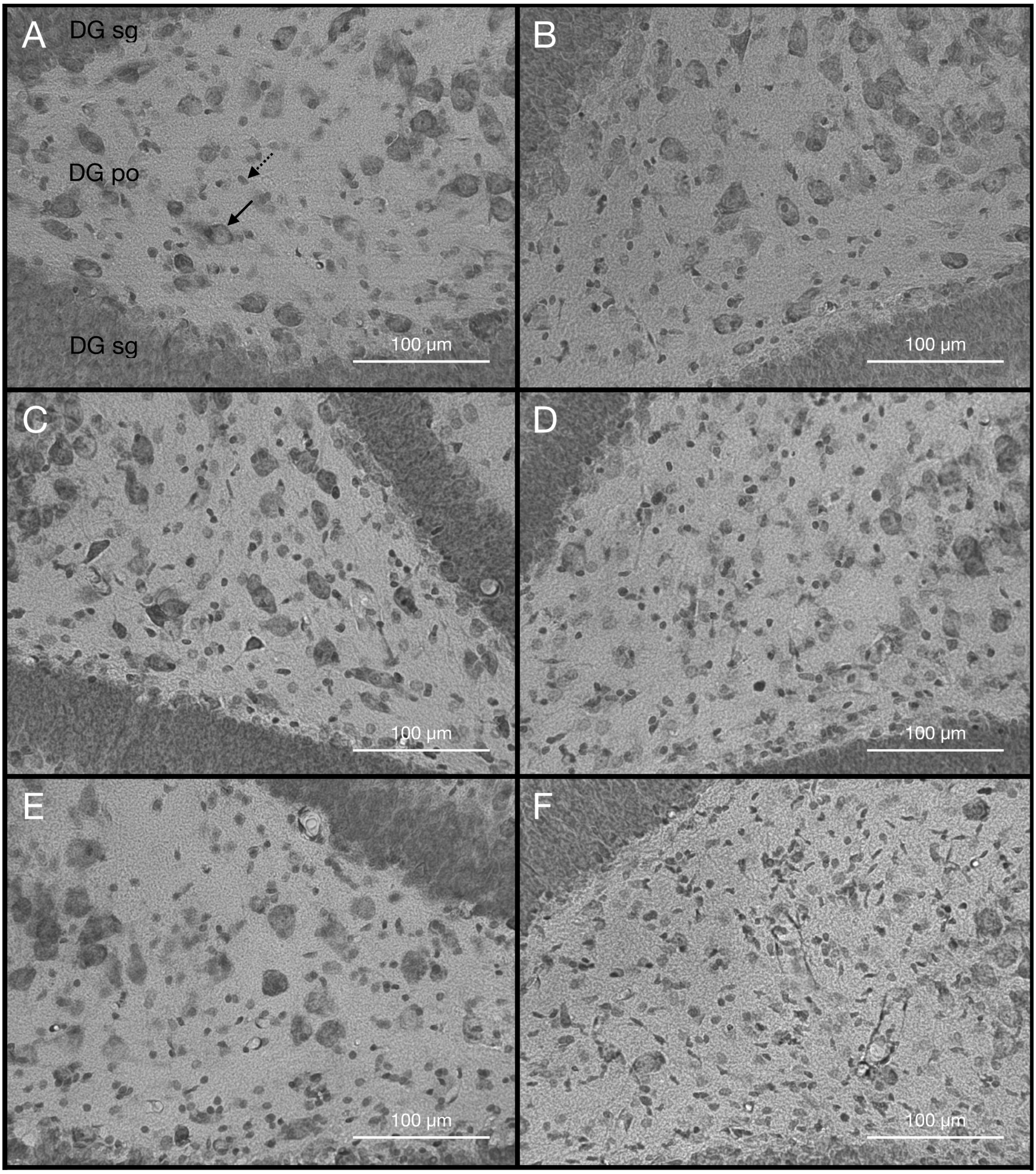

Figure S5. Neuronal cell loss in the hippocampus. A – control, right hemisphere. B – Sham operated rats, day 7 after craniotomy. C, D – contralateral and ipsilateral hippocampus (ipsilateral cell loss), day 3 after TBI. E, F - contralateral and ipsilateral hippocampus, day 7 after TBI (bilateral cell loss). Nissl staining. DG – dentate gyrus, sg – granular cell layer, po – polymorph layer. Solid arrow shows neurons, dotted arrow shows nucleus of glial cell.

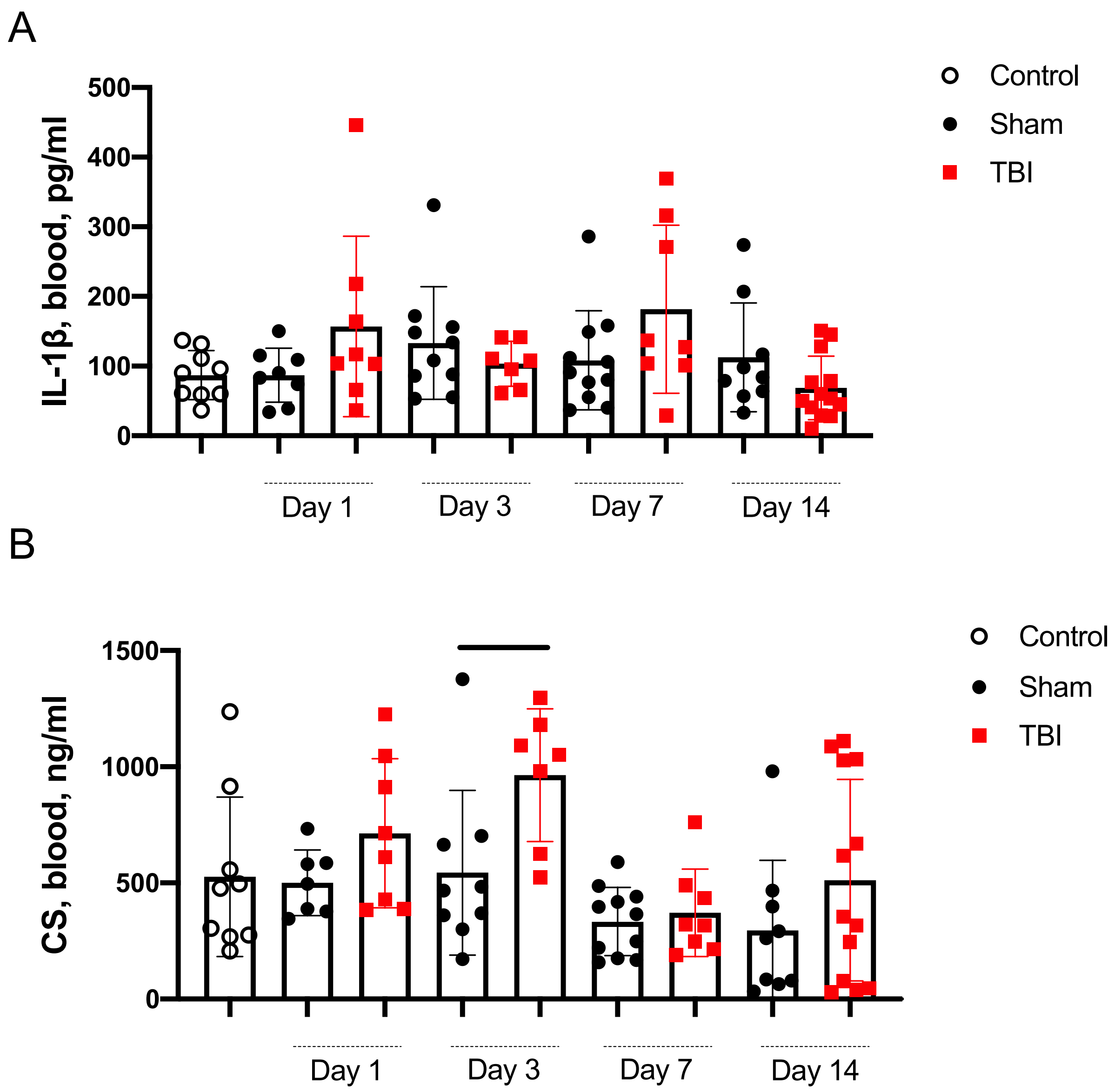

Figure S6. IL-1β and CS in blood. A – IL-1β in blood does not change significantly after TBI. B – CS in blood is elevated on day 3 after TBI.
